# The evolution of pre-descemet endothelial keratoplasty (PDEK)

**DOI:** 10.1038/s41433-026-04655-2

**Published:** 2026-07-28

**Authors:** Matteo Ripa, Krystal Gayle, Dalia G. Said, Harminder S. Dua

**Affiliations:** 1 Department of Ophthalmology, Queen’s Medical Centre, Nottingham, UK; 2 Academic Ophthalmology, Mental Health and Clinical Neurosciences, School of Medicine, University of Nottingham, Nottingham, UK

**Keywords:** Transplantation, Outcomes research

## Abstract

Pre-Descemet’s endothelial keratoplasty (PDEK) is an evolving endothelial keratoplasty (EK) procedure that merges the advantages of Descemet membrane endothelial keratoplasty (DMEK) and ultrathin EK methods. This review reports the latest anatomical, surgical, and clinical research on PDEK and discusses its potential future role in current EK practices. Anatomical data show that the Pre-Descemet/Dua’s Layer (PDL/DL) acts as a biomechanical support, or “splint,” for Descemet’s Membrane (DM). This support facilitates the creation of grafts that are less tight and easier to handle compared to DMEK tissue. Notably, PDEK leads to rapid visual recovery and improvements in best-corrected visual acuity after surgery. Since PDEK results in an acceptable level of endothelial cell loss and has demonstrated reasonable detachment and rebubbling rates in published series, particularly in experienced hands, it is regarded as a reliable method for effectively utilising young and paediatric corneas, thereby optimising tissue use in eye bank procedures. Furthermore, advancements in surgical techniques, such as specialised PDEK clamps, an air-pump-assisted method, consistent 360-degree scoring, and the integration of optical coherence tomography into the surgical microscope, have resulted in higher success rates in creating Type 1 big-bubble, decreased overall tissue loss, and enhanced procedural consistency. Finally, PDEK merges the optical advantages of DMEK with enhanced intraoperative control and expanded donor options with the inclusion of young donor eyes, and is likely to gain wider adoption when integrated into training programs and eye bank protocols.

## Introduction

Since 1931, when Vladimir Filatov successfully performed full-thickness corneal transplants in humans, corneal transplantation surgery has evolved remarkably with continuous refinement. For decades, penetrating keratoplasty (PK) has remained the gold standard for managing corneal opacities and endothelial failure [1]. However, over the years, the limitations of full-thickness corneal replacement, such as delayed epithelialisation, slippage of graft host wound edges, unpredictable astigmatism, immunological rejection, suture-related complications, and failure have prevailed despite advances in surgical techniques, instrumentation, and medication [2]. Following Eduard Konrad Zirm’s first successful partial-thickness human corneal transplant, after a lapse of many years, lamellar keratoplasty has re-emerged as a forerunner in the field of corneal transplantation. It offers the replacement of diseased corneal layers while preserving healthy host tissue. Today, endothelial keratoplasty (EK), involving the targeted replacement of the diseased corneal endothelium and Descemet membrane (DM), is the gold standard for conditions characterised by DM and endothelial cell dysfunction. This paradigm shift, involving Descemet stripping automated endothelial keratoplasty (DSAEK) and Descemet membrane endothelial keratoplasty (DMEK), mitigated or eliminated many of the complications associated with full-thickness grafts, including intraoperative devastating suprachoroidal haemorrhage, or rupture of the globe with trivial trauma, even several years postoperatively. At the same time, it considerably shortened the time to visual recovery and increased graft survival [3]. The discovery of the pre-Descemet layer/Dua layer (PDL/DL) led to the development of Pre-Descemet Endothelial Keratoplasty (PDEK). This technique combines the visual benefits of DMEK and ultrathin DSAEK, with technical advantages in performing surgery and minimising endothelial cell loss and enabling the use of a wider donor age range, thus providing an additional dimension to the ongoing progress of EK [4]. Despite an expanding body of literature describing this evolving surgical technique [5–34], to our knowledge, a comprehensive, focused review on the translational aspects and evolution of PDEK is lacking. The purpose of this review was to summarise the evolution, rationale, and clinical outcomes of PDEK, with particular emphasis on its advantages and future role in corneal transplantation.

## Materials and methods

Although a narrative design was employed for this review, a structured literature search was performed to enhance methodological rigour and ensure that all relevant publications on the anatomical rationale, surgical techniques, and clinical outcomes of PDEK were included. Three databases (Scopus, PubMed, and Embase) were analysed as of 20 September 2025. For the literature retrieval process, the search strategy included the terms ‘PDEK’ OR ‘Pre-Descemet Endothelial Keratoplasty’ to identify all studies reporting surgical techniques, clinical outcomes, or anatomical insights directly related to PDEK. After completing the initial database search, a hand-search evaluation of the reference lists of all included articles was conducted to identify additional relevant studies that had not been previously identified.

We included all original studies, but excluded literature reviews, dissertations, book chapters, and conference abstracts that reported on various aspects of PDEK. Articles that merely cited the PDEK or focused exclusively on other forms of endothelial keratoplasty (e.g., DMEK, DSAEK) were also excluded. In addition, reports not available in English were excluded. After duplicate removal, 27 papers were selected for the current review from an initial search yielding 218 records (62 from PubMed, 77 from Embase, and 79 from Scopus). The hand-search bibliography evaluation of all included articles identified two additional articles, bringing the total to 29 studies.

## Surgical technique of PDEK

The concept of PDEK was introduced in 2013 by Dua et al. to define a tissue composite of PDL/DL, DM, and endothelial cells (EC) for EK treatment of patients with corneal endothelial pathology. In 2014, Agarwal A. and Dua H.S. published the first report on the PDEK surgical technique in five eyes of five patients, with successful graft attachment and good postoperative visual recovery in all cases. However, on retrospective review, case reports of ‘DMEK’ have been published before, where the surgeon had in fact performed PDEK. Over the last ten years, the original technique has been widely adopted, and many refinements have been introduced to further optimise and standardise the procedure [24].

In the following sections, we outline the key steps of the procedure, including donor tissue preparation, recipient bed preparation, donor lenticule implantation, unfolding and positioning, tamponade, postoperative management, and multiple or combined procedures.

### Donor tissue preparation

#### Standard technique

The donor tissue is commonly prepared by adapting the big-bubble (BB) technique on donor corneo-scleral discs as described by Dua et al. [4], which involves the creation of a type 1 BB to separate the PDL/DL, DM, and endothelium from the overlying stroma and trephining or excising the bubble wall for transplantation as reported by Agarwal and Dua [24]. This technique has become the standard approach, with several modifications made to enhance safety, reproducibility, and surgical outcomes [24]. The tissue preparation aims to achieve a type 1 BB as large as possible in the donor cornea to obtain a composite of the PDL/DL, DM, and EC. To accomplish this, air is injected via a 30-gauge needle connected to a 5 mL air-filled syringe. A corneo-scleral disc, which has an approximate 2 mm scleral rim, is placed endothelial side up, on a suitable sterile base such as the back of a Gallipot. A black metal plate has been made to provide better contrast and reduce reflections. Under the operating microscope, the needle, bevel up, bent to 45 degrees, is inserted through the stroma of the scleral rim and advanced to the mid-periphery of the posterior corneal stroma. Air is injected continuously until the BB starts to appear by the coalescence of several small bubbles, when the force on the plunger is reduced but maintained until the BB ceases to expand. If a large bubble, approximately 9 mm in diameter, forms, it can be deflated by inserting the needle tip into the bubble and drawing out the air. Equally, after advancing the needle tip in the bubble through the posterior stroma, the BB can be inflated gently to achieve maximum diameter. The wall of a deflated large BB can be punched with a donor punch of appropriate diameter (7–8 mm) or excised with a pair of Vannas scissors along the circumference of its attachment to the posterior stroma. To do this, the bubble wall is punctured with a fine lance blade at the outer edge, and trypan blue dye is injected into the bubble cavity to stain the graft, which is then cut along the entire circumference. When lifting the PDEK graft tissue off the posterior stroma, fine attachments in the form of collagen strands may be seen and can be cut [24].

#### Technical variations and refinements

Several donor bubble-dissection methods have been proposed to enhance PDEK lenticule preparation by achieving type 1 BB formation decreasing the chances of type 2 BB formation, and reducing risk of preparation failure. These methods and approaches have been guided by the defined peripheral anatomical characteristics of the PDL/DL and include pressure-control system, circumferential scoring technique, imaging-guided method, and specialised preparation devices.

Determined by the peripheral anatomy of the PDL/DL, the injected air may escape through the clusters of holes located in the periphery of the PDL/DL along the circumference of the cornea [35]. This allows air to access the plane between the PDL/DL and DM, resulting in the lifting of the DM and the formation of a type-2 BB, thus creating tissue suitable for DMEK. To avoid this undesirable outcome, in 2016, Dua et al. created a surgical clamp consisting of a spring handle with two complementary rings and a central locking screw to hold the clamp closed, the “PDEK clamp”, to shut all the peripheral fenestrations and prevent escape of air to allow the air to only accumulate in the donor corneal stroma and facilitate the formation of a type 1 BB [7]. The clamp, by preventing any loss of air, enables a controlled rise of intrastromal pressure to attain a level sufficient to create a type 1 BB. The PDEK clamp is available for right-handed and left-handed surgeons [18].

To mitigate the risk of type 2 BB, in 2019, Saint-Jean et al. proposed a new technique, the “Soper technique.” It involved a 360° scoring of the DM and endothelium just inside the Schwalbe’s line, followed by incremental air injection to provide an escape route for injected air and avoid type 2 BB formation, achieving a type 1 BB in 24 of 26 attempted cases (92.3%) [16]. This is a slight variation of the technique described by Pereira et al. [26]. Specifically, they described a technique useful for avoiding the air to access the plane anterior to the DM, preventing the formation of a type 2 BB. They scored the DM circumferentially along the stromal periphery, forming an escape route for air emerging from the fenestrations, achieving type 1 BB formation in 100% of corneas [26]. However, this technique does not enable a controlled increase in intra-tissue pressure, which is an advantage of the PDEK clamp described earlier.

In 2022, Agarwal et al. proposed the use of the intraoperative (i)-optical coherence tomography (OCT) to guide the donor preparation, to evaluate the depth of the needle, orientation, and direction of the bevel before air injection. They performed four PDEK tissue preparations under i-OCT guidance, finding that in cases of multiple air bubbles, the i-OCT could guide precise needle advancement for successful bubble expansion. Additionally, during donor excision with scissors, i‑OCT guidance could assist in removing strong residual adhesions [19].

Recently, Kalinnikov et al. presented a revised system comprising a dedicated base, ring fixator, and spring-loaded syringe. In their series, type 1 bubble formation occurred in all cases (10/10) with the new method, compared to only half (5/10) with the standard approach. Bubble rupture and type 2 bubble formation were entirely prevented (0%) with the new method, whereas they occurred in 30% and 20% of cases, respectively, using the standard approach. Importantly, this technique also enabled the preservation of PDEK grafts for eye banking, thereby reducing tissue waste and expanding the procedure’s translational potential.

Although these approaches share the common objective of improving successful type 1 BB formation and reducing preparation failure, direct comparative evidence between techniques remains limited. Therefore, the selected technique may vary based on surgeon preference, available equipment, and the desired balance between simplicity and procedural control.

### Recipient bed preparation

In the recipient eye, PDEK requires the removal of DM and endothelium by performing a complete Descemetorhexis. In accordance with other EK procedures, preparing the recipient begins with creating an epithelial mark corresponding to the area of the DM to be removed, using an appropriately sized trephine or other marker. After creating a 2.8 mm corneal tunnel incision, a reverse Sinskey hook is inserted into the anterior chamber (AC). A circular section of DM is then carefully scored using the epithelial mark as a guide. The scored DM can be peeled or stripped off the posterior surface of the PDL/DL. This is a very gentle procedure that ensures the PDL/DL is not abraded. In cases with long-standing corneal oedema, the PDL/DL can be inadvertently peeled/pulled away from the posterior stroma during this step of the procedure and should be avoided. The edge of the DM can be grasped with a non-toothed forceps and peeled off in a capsulorhexis-like manner, before removal [24]. If Descemetorhexis is incomplete, tags of the DM may remain around the edge. These can be carefully held with forceps and peeled away, as they could hinder graft attachment. Alternatively, they can be sucked into the port of an aspiration cannula and removed. Free fragments attached to the posterior stroma (recipient PDL/DL) can also be similarly aspirated out. Trypan blue can be injected in the AC to visualise any DM remnants. Alternatively, the AC can be filled with air, which enhances contrast and makes it easier to visualise the edges of residual fragments and tags [18]. Jacob et al. enhanced this step by using a pressurised air infusion system via an anterior chamber maintainer, enabling controlled inverse descemetorhexis and improved visualisation under air. To prevent postoperative pupillary block, they recommended performing a small inferior peripheral iridectomy with a vitrector [17].

### Donor PDEK tissue implantation

The donor tissue, stained with trypan blue, is inserted in the AC using a lens injector modified by removing the spring on the piston. Removing the spring avoids suction and inadvertent damage to the donor tissue, as previously suggested by Price [36], and applied by Agarwal et al., in PDEK [24]. Ross et al. reported further improvements and refinements in a step-by-step approach that could be used for both scroll and trifold preparations. These include using alternative delivery techniques, such as pulling the graft into the AC with Busin forceps, keeping a column of balanced salt solution to avoid air bubbles, and carefully orienting the tissue inside the cartridge [18]. The PDL/DL surface of the PDEK tissue can be marked with an asymmetrical alphabet (S, F or P).

### Unfolding and positioning

The same unfolding technique commonly used for DMEK can be applied to PDEK tissue, but it is easier with PDEK because the tissue scrolls less. In an ex vivo study, Dua et al. confirmed this feature by comparing the scrolling behaviour of PDEK tissue with its individual parts. They first graded the scroll of PDEK tissue, then separated the DM from the PDL/DL and graded the scroll of each of these. When compared to the DM alone and the PDL/DL alone, they found that PDEK tissue had an intermediate scrolling level, suggesting that the PDL/DL acts as a “splint,” reducing the DM’s tendency to scroll. Less scrolling makes surgical handling easier and unscrolling in the anterior chamber more predictable [9]. In a relatively flat AC, tapping the corneal epithelium from outside forces the fluid between the folds, thus pushing them outward, and results in the folds opening. Simultaneously, the proximity of the posterior cornea to the iris keeps the tissue edges in place without recoiling until repeated tapping fully opens it. Graft orientation is verified by the alphabet mark, observing the orientation of the scroll in the AC before tapping, or with an i-OCT ensuring that the outside of the scroll stays posteriorly directed (for scrolled tissue) or the opposite for trifold tissue. In case of poor visualisation due to corneal oedema, the epithelium may be removed, and/or an endoilluminator can be employed externally, directed into the AC from the limbus, to facilitate the visualisation of the graft within the AC [19].

Jacob et al. recently introduced an air pump-assisted PDEK technique that employs continuous air infusion to maintain AC stability during graft handling. The correctly oriented and opened PDEK graft is apposed to the posterior surface of the host PDL/DL with air and a reverse Sinskey hook is used to engage the edge of the graft and pull it in the desired direction for precise centration. The hook is also used to gently tease open peripheral folds in the graft, thus preventing endothelial cells on the fold from apposing against the posterior surface of the recipient PDL/DL and potentially reduce the risk of graft dislocation [17]. Jacob et al. reported that continuous air infusion aids in centring the graft, unfolding peripheral edge folds, and smoothing creases, effectively acting as a ‘third hand’ inside the AC [13].

### Tamponade and postoperative management

After unfolding and correctly positioning the PDEK tissue, a Rycroft cannula (Sterimedix Ltd., Redditch, UK), attached to a 2 mL syringe filled with air (or SF6 20%), is gently inserted through a side port between the donor tissue and the iris. It is carefully slid along the iris, avoiding contact with the graft’s endothelial surface. Once the cannula tip reaches the centre of the pupil, air is slowly injected to elevate the graft and gently press it against the posterior surface of the cornea. The plunger’s smooth movement should always be checked in advance to avoid unexpected over-injection and/or graft displacement. A paracentral injection may cause the graft to be elevated asymmetrically, resulting in decentration. In such circumstances, the tissue can be gently adjusted by stroking the PDL/DL surface with a blunt spatula or pulling with the point of a 30 gauge needle bent upwards or with a reverse Sinskey hook as described above, before completing the air fill. The AC is subsequently filled with air/gas to about 30 mmHg and held for about 10 min before gradually releasing the bubble to maintain a near-full air fill, to prevent excessive intraocular pressure (IOP). In cases of wound leakage, side ports may be hydrated or sutured, and additional air can be injected directly via a 30-gauge needle through a limbal puncture. For a longer tamponade effect, a 20% SF6 mixture in air may be employed. Finally, to reduce the risk of pupillary block glaucoma, a preventive inferior peripheral iridectomy is helpful. This can be performed preoperatively with a YAG laser or intraoperatively, albeit the latter may raise the risk of haemorrhage and fibrin development in the AC [18, 24]. The patient should remain in a supine position for two hours to facilitate graft attachment. IOP must be monitored at one and two hours following the procedure. If the IOP measures between 21 and 30 mmHg, administration of oral acetazolamide tablet (500 mg) should be considered, alongside pupil dilation with cyclopentolate 1% and phenylephrine 2.5%. Higher IOP can be managed with intravenous acetazolamide or by releasing air from the AC, preferably the latter. Graft attachment can be assessed clinically by slit lamp examination and by anterior segment (AS)-OCT, facilitating early detection of small pockets of graft detachment. In addition to confirming graft adherence, AS-OCT can identify graft inversion. Kumar et al. described the “mouse sign” observed on AS-OCT, characterised as a convex detached scroll with one edge adherent to the host cornea. This phenomenon may be associated with uncorrected rolled or curled edges at the conclusion of surgery, inadequate intraocular air support, inverse graft positioning, stromal irregularities, or incomplete removal of recipient DM [11]. Postoperative medications include a steroid (dexamethasone 0.1%) six times a day for one week, tapering to four times a day for one month, and then gradually. An antibiotic (either chloramphenicol or fluoroquinolone) is used four times daily for 2–4 weeks, and a mydriatic (cyclopentolate 1%) twice daily for three days [18].

### Multiple/combined procedures

In complex anterior segment diseases, PDEK has also been effectively combined with other surgical procedures to address multiple issues in a single session. Kumar et al. reported a new triple procedure of phacoemulsification, pinhole pupilloplasty, and PDEK for the treatment of endothelial decompensation and irregular astigmatism simultaneously [28]. Narang et al. reported the use of glued intraocular lens (IOL) fixation combined with pupilloplasty and PDEK in aphakic or IOL-malpositioned eyes to maintain chamber stability and achieve effective air tamponade [5, 21, 25]. They later developed the double-infusion cannula technique, which combines posterior fluid infusion with anterior pressurised air infusion to facilitate glued IOL fixation and PDEK, while reducing intraoperative fluctuations and enhancing graft stability and adhesion [33]. A successful triple procedure involving glued IOL fixation, single-pass four-throw pupilloplasty, and PDEK was recently described in a patient with a history of pseudophakic bullous keratopathy (PBK) following complicated cataract surgery [34].

## Clinical outcomes

Since the first publication of the PDEK procedure [24], several clinical studies have further confirmed its efficacy (Table 1). In Amar Agarwal’s five-eye case series, all surgeries succeeded, despite one graft detachment in a single quadrant with a shallow anterior chamber. A significant postoperative decrease in central corneal thickness (CCT) and a substantial improvement in best-corrected visual acuity (BCVA) after surgery were reported. In the final follow-up, 4 of 5 patients achieved a BCVA of 20/30, while one patient reached 20/40. The mean endothelial cell density post-surgery was 1551.2 ± 65.2 cells/mm² [24]. In 2015, Ashvin Agarwal et al. used infant donor cornea to partly compensate for the endothelial cell loss (ECL) during PDEK and keep the postoperative endothelial cell density (ECD) at a high level. In their case series of 3 PDEK procedures, they reported an ECL of 27 ± 2% 6 months postoperatively [30]. In 2017, Amar Agarwal et al. evaluated the role of young donor tissue in chronic PBK, combined with epithelial debridement which facilitated clear visualisation and easier graft handling. Of their six eyes, four were treated with PDEK. They used donor corneas from infants to young adults (aged 9 months to 33 years), follow-up between 6 and 18 months revealed notable improvements in vision with PDEK (up to 20/40) and a decrease in CCT, and acceptable ECL (36.5 ± 10.4%). There were no instances of rebubbling, graft detachment, rejection, or failure. Therefore, when PDEK is combined with epithelial debridement utilising young donor corneas, it can be effectively performed even in eyes exhibiting long-standing corneal oedema and anterior stromal scarring [12].

**Table 1:** Characteristics and outcomes of clinical studies on PDEK.

First Author	Year	Eyes (n)	Patients Age (mean / range) Patients Gender	Indication (%)	Donor Age (mean / range)	Technique Variation (e.g., air-pump, Soper, i-OCT, combined)	Follow-up (days/months mean ± SD)	Preop BCVA (Decimal/Snellen/LogMar, mean ± SD) Postop BCVA (Decimal/Snellen/LogMar, mean ± SD)	Preop CCT (µm, mean ± SD) | Postop CCT (µm, mean ± SD)	Donor Preop ECD (cells/mm²) | Host Postop ECD (cells/mm²)	% loss of ECD (mean ± SD) Graft Size (mm, mean ± SD) Graft Thickness (µm, mean ± SD)	Rebubbling Rate (%) Graft Detachment Rate (%) Graft Failure (%)
Agarwal et al. [24]	2014	5	/ /	/	/	PDEK	30.2 ± 0.8	/ 0.62 ± 0.07	707.2 ± 88.7 /	/ 1551.2 ± 65.2	/ 7.6 ± 0.22 38.4 ± 5.3	20 20 0
Agarwal et al. [30]	2015	3	60 ± 16.1 Male: 2 Female: 1	PBK	/	PDEK: 2 PDEK + IOL explantation + fixation of a 3-piece IOL: 1	11 ± 4.6	0.1 ± 0.1 0.5 ± 0.4	/ 515 ± 7	3073 ± 68 2230 ± 43	27 ± 2 / /	0 0 0
Agarwal et al. [12]	2017	4	Patient 1:73 Patient 2:56 Patient 3:68 Patient 4:64 Patient 1: Male Patient 2: Male Patient 3: Female Patient 4: Female	Patient 1: PBK + decentred PC IOL Patient 2: PBK + PC IOL Patient 3: PBK Patient 4: PBK	Patient 1:9 Patient 2:33 Patient 3:20 Patient 4:18	Patient 1: Glued IOL + PDEK Patient 2: Glued IOL + PDEK Patient 3: PCIOL, PDEK Patient 4: PCIOL, PDEK	Patient 1:18 Patient 2:9 Patient 3:6 Patient 4:6	Patient 1:FC Patient 2:FC Patient 3:FC Patient 4:FC Patient 1: 20/120 Patient 2: 20/40 Patient 3: 20/200 Patient 4: 20/80	Patient 1:640 Patient 2:560 Patient 3:788 Patient 4:592 534.6 ± 21.1	Patient 1: 2996 Patient 2: 2255 Patient 3: 3169 Patient 4: 3230 1475.7 ± 444.6	36.5 ± 10.4 Patient 1: 8.3 ± 0.5 Patient 2: 8.3 ± 0.5 Patient 3: 8.3 ± 0.5 Patient 4: 8.3 ± 0.5 / / / /	/ / /
Jacob et al. [17]	2017	10	/	PBK: 8 FECD with cataract: 1 ICE: 1	<40	Air pump–assisted PDEK: 8 Air pump–assisted PDEK + synechioloisis: 2	6	0.11 ± 0.09 0.66 ± 0.25	/ /	2820 ± 674 1730 ± 265	38.6 / /	10 20 /
Narang et al. [21]	2017	9	/ /	Iris defect + Endothelial Decompensation	/	SFT Pupilloplasty + PDEK ( ± glued IOL)	/	/ /	/ /	/ /	/ / /	0 0 0
Kumar et al. [11]	2018	3	Patient 1:60 Patient 2:64 Patient 3:59 Patient 1:Female Patient 2:Female Patient 3:Male	Patient 1:PBK Patient 2:PBK Patient 3:PBK	Patient 1:32 Patient 2:19 Patient 3:47	Patient 1: PDEK Patient 2: PDEK ( + AC-IOL explant, glued IOL, pinhole pupilloplasty) Patient 3: PDEK	/ / /	Patient 1: 0.01 Patient 2: FC Patient 3: 0.3 / / /	/ / / / / /	/ / / / / /	/ / / Patient 1:7.5 Patient 2:7 Patient 3:7 Patient 1:24 Patient 2:36 Patient 3:48	100 100 100
Jacob et al. [13]	2018	12	/ /	PBK: 10 ICE: 1 Viral Uveitis: 1	/	Air pump–assisted PDEK: 5 Air pump–assisted PDEK + synechioloisis: 4 This air pump–assisted PDEK + pupilloplasty:4	6	0.13 ± 0.13 0.62 ± 0.31	/ /	3033 ± 74 1916 ± 309	/ / /	16.6 16.6 0
Narang et al. [34]	2018	1	23 Female	PBK	/	Phaco + SFT Pupilloplasty + PDEK	6	20/800 20/20	/ /	3250 2595	20.15 / /	0 0 0
Narang et al. [25]	2019	25	/ /	PBK	35.1 ± 10.4	Glued IOL + SFT + PDEK	≥12	1.51 ± 0.76 0.53 ± 0.53	/ /	/ 2207 ± 59.6	31.9 ± 5.93 7.6 ± 0.9 /	0 0 0
Kumar et al. [23]	2020	21	56.4 ± 16.2 Male: 10 Female: 10	PBK	/	PDEK	17.7 ± 17	1.31 ± 0.46 0.11 ± 0.12	/ 502 ± 45	/ 1389 ± 457	/ 7.6 ± 0.64 /	/ / /
Sharma et al. [19]	2021	4	45 ± 24 (18–74) Male: 2 Female: 2	PBK: 3 FECD: 1	/	i-PDEK (microscope-integrated OCT assisted)	6	FC: 2 eyes 0.78: 1 eye 1.48: 1 eye 0.6: 2 eyes 0.3: 2 eyes	747.5 ± 17.1 557.3 ± 13.5	/ 1736 ± 42	21.6 ± 0.02 7.75 ± 0.20 25.5 ± 2.64	0 0 0
Nidhi et al. [8]	2022	43	68 ± 9.6 Male: 25 Female: 18	PBK: 35 ABK: 5 FECD: 3	/	PDEK alone: 33 PDEK ± IOL explantation + secondary IOL implantation: 10	/	1.41 ± 0.33 0.29 ± 0.12	653.1 ± 96 524.8 ± 45	/ 1596 ± 392	/ / /	/ / /
Kumar et al. [28]	2023	2	/	KC + FECD	/	Phacoemulsification with IOL implantation, + PPP, + PDEK	6	BE: 20/80 BE: 20/30	/ RE: 531 LE: 518	/ /	/ / /	0 0 0
Narang et al. [10]	2024	12	50.25 ± 12.79 (22–68) Male: 9 Female: 3	Endothelial decompensation after failed therapeutic PK	25.25 ± 5.86	PDEK alone: 3 PDEK + phaco: 6 PDEK + pupilloplasty: 3	18.5 ± 4.9	0.02 ± 0.01 0.54 ± 0.17	/ /	3271 ± 175 2338 ± 288	28.2 ± 10.6 7.63 ± 0.68 /	8.3 8.3 /

*BCVA* best corrected visual acuity, *CCT* central corneal thickness, *ECD* endothelial cell density, *EK* endothelial keratoplasty, *PBK* pseudophakic bullous keratopathy, *Mo* months, *d* days, *SD* standard deviation, *n* number, *i-OCT* intraoperative- optical coherence tomography, *ICE* iridocorneal endothelial syndrome, *PK* penetrating keratoplasty, *ABK* Aphakic Bullous Keratopathy, *FECD* Fuchs Endothelial Corneal Dystrophy, *PC* posterior chamber, *FC* fingers counting, *KC* keratoconus, *PPP* pinhole pupilloplasty, *BE* both eyes, *RE* right eye, *LE* left eye, *SFT* singlepass 4-throw, *AC-IOL* anterior chamber intraocular lens, *PCIOL* posterior chamber intraocular lens, *Phaco* phacoemulsification, *LogMAR* logarithm of the minimum angle of resolution, IOL intraocular lens.

Beyond the original technique described by Amar Agarwal et al., several authors have advocated the use of further tools to facilitate PDEK. In 2017, Jacob et al. proposed an air pump-assisted PDEK technique that employed continuous pressurised air infusion to maintain AC stability during graft handling. In the 10 patients who underwent surgery, at 6 months the BCVA improved significantly from 0.11 ± 0.09 to 0.66 ± 0.25, while ECD decreased from 2820 ± 674 to 1730 ± 265 cells/mm², corresponding to a 38.6% loss. Two partial detachments required rebubbling, and one case was vision-limited by myopic maculopathy, but all corneas remained clear [17]. Furthermore, in another case series of 12 patients, where this technique was used, Jacob reported that all grafts were clear at 6 months with mean BCVA improving significantly from 0.13 ± 0.13 to 0.62 ± 0.31 and ECD decreasing from 3033 ± 74 cells/mm² to 1916 ± 309 cells/mm² ( ≈ 36% loss). Two cases of partial detachment occurred (one resolved spontaneously, while the other required rebubbling). The technique stabilised intraoperative chamber conditions, assisted descemetorhexis and graft unfolding, and improved early adhesion. The authors noted that continuous pressurised air infusion acted as a “third hand” in the AC, aiding centration, edge unfolding, and uncreasing tightly scrolled grafts [13].

In 2021, Sharma et al. reported the first results of using the i-OCT to guide the various steps of the procedure. They found that the i-OCT could facilitate successful type 1 BB formation during donor preparation, debridement of the hypertrophic epithelium, planning and placement of surgical wounds, descemetorrhexis with removal of remnant DM tags, and identification of correct donor orientation and interface details. In their case series of 4 patients, type 1 BB was successfully formed in all cases, and at 6 months, all grafts remained clear and well attached. The average CCT was 557.25 ± 13.45 µm; graft diameter was 7.75 ± 0.20 mm; graft thickness was 25.5 ± 2.64 µm; and ECL was ≈ 21.6% [19].

Beyond assessing visual acuity and endothelial cell survival, the optical quality after PDEK has also been studied. In 2020, Kumar et al. measured corneal backscattering post-PDEK using densitometry from Scheimpflug imaging and compared it with the fellow normal eyes. They observed that eyes with a BCVA of 20/20 showed no significant difference in total corneal densitometry across the entire corneal depth between PDEK and control eyes, indicating that PDEK eyes can replicate the optical properties of near-normal corneas. Their cohort study revealed that densitometry in the central 0- to 2-mm and 2- to 6-mm zones was lower than in the peripheral annulus. Moreover, the anterior corneal layers exhibited higher backscattering overall, while the posterior layers showed the least. Interestingly, densitometry measurements in the 0 to 2 mm and 2 to 6 mm zones after PDEK correlated with BCVA, highlighting the importance of central corneal backscattering in determining final visual outcomes [23].

Although multiple studies have reported visual acuity after PDEK, in 2022, Nidhi et al. first compared changes in the posterior corneal curvature following PDEK and related these changes to visual outcomes. They determined that the donor-to-host curvature mismatch did not result in any permanent, significant alterations and that the substantial improvement in BCVA from before surgery to after was not related to changes in overall corneal power or posterior corneal curvature. PDEK caused a temporary flattening of the posterior cornea, which normalised by 6 months, indicating refractive stability. The average posterior radius decreased from about 6.52 mm before surgery to 6.11 mm at 1 month, then gradually increased to around 6.44 mm at 6 months. Meanwhile, the overall corneal power remained essentially unchanged. This pattern suggests that as the graft attaches and the stroma dehydrates, the posterior surface reverts to its preoperative shape, accounting for both stable refraction and the improvements in BCVA [8].

Despite higher success rates reported in several studies, Kumar et al. in 2018 described three cases of patients who had previously undergone uneventful PDEK with less than 2 weeks of graft detachment. Their AS-OCT images resembled a computer mouse. After a repeat air injection, the graft detachments reoccurred with the same pattern. All three patients exhibited a similar pattern: partial attachment at one end, followed by outward, downward, upward, and inward folding of the DM, forming that shape. However, in the image illustrated in the paper, the PDEK graft seems to have been placed upside down, as shown by the scroll, which should have been in the other direction if the graft was placed in the correct orientation, as we know that the PDEK tissue, like DMEK tissue, scrolls with the endothelial cells on the outside. Nevertheless, the mouse sign would still be apparent. Several possible causes, such as uncorrected rolled or curled edges at surgery, insufficient intra-chamber air support, or inverse graft placement, were considered. Still, no subsequent reports have documented similar postoperative graft configurations [11].

PDEK has also been performed in patients who had previously undergone failed PK. In 2024, Narang et al. reported the outcomes of PDEK in 12 eyes that had previously undergone therapeutic PK. Following an average follow-up of 18.5 months, BCVA improved significantly from 0.02 ± 0.01 to 0.54 ± 0.17, with 67% of eyes achieving 20/30 or better vision. Corneal clarity was restored in most cases, with 9 eyes graded as completely clear. ECD decreased from 3271 ± 175 to 2338 ± 288 cells/mm², representing an average cell loss of 28.2%. The complications included one case of partial graft detachment requiring rebubbling, one instance of successfully treated immune rejection, and one case of secondary glaucoma needing a valve implantation. Narang et al.’s findings suggest that PDEK may offer optical stabilisation and reasonable survival rates, even in challenging situations of failed therapeutic PK, thereby expanding its potential uses beyond primary endothelial failure [10].

Many authors have reported satisfactory outcomes with PDEK alone or with multiple surgeries in a single sitting. In 2018, Narang et al. performed a triple procedure involving phacoemulsification, single-4-throw (SFT) pupilloplasty, and PDEK in a patient’s eye that had suffered a complication of a cosmetic iris implant. The patient attained 20/20 vision postoperatively [25]. In the same year, Narang et al. described a combined triple procedure of glued IOL, SFT pupilloplasty, and PDEK for PBK. They found this triple procedure was an effective option to optimise the visual outcome in cases with endothelial dysfunction and inadequate posterior capsule support. Specifically, they noted that performing the glued IOL first is essential to ensure proper IOL fixation, followed by SFT pupilloplasty. This sequence provides stability to the anterior chamber and prevents air from entering the vitreous cavity, thereby facilitating the eventual PDEK procedure [34]. In 2023, Kumar et al. performed a triple procedure in both eyes of a patient affected with KC and FECD, performing phacoemulsification, pinhole pupilloplasty, and PDEK. After surgery, unaided vision improved to 20/30 in both eyes [21].

## Comparisons and challenges

Among modern EK options, PDEK has been proposed to combine the visual potential of DMEK with easier intraoperative handling due to the inherent anatomical attributes of the PDL/DL layer. Nonetheless, only a few reports comparing the outcomes of both techniques have been published. In 2022, Shanmugam et al. found that the two approaches were equivalent in visual quality at 6 months, and that BCVA, contrast sensitivity, and higher-order aberrations improved similarly in patients undergoing surgery for endothelial decompensation. However, they diverged anatomically: DMEK resulted in a greater CCT reduction ( ≈ 196 ± 26 µm vs 140 ± 14 µm) and a thinner graft–host complex at 6 months ( ~ 16 µm vs ~27 µm for PDEK). The difference corresponds to the thickness of the PDL/DL. Despite these differences in thickness, endothelial survival was similar, with an ECD loss of 35% for DMEK and 33.4% for PDEK. The complication profiles were similar, but rebubbling was higher after DMEK (3/15, 20%) compared to PDEK (1/15, 6.7%), although the difference was not statistically significant. Intraoperatively, surgeons observed that PDEK enabled quicker and easier donor preparation with less intracameral manipulation, thanks to the “splinting” effect of PDL/DL on DM. In contrast, DMEK maintained the highest anatomical accuracy but required more stringent donor criteria and greater technical skill [22].

A challenging issue pertains to whether or not very young or infant donor tissue should be utilised. PDEK has specifically explored this niche: Agarwal and colleagues reported on using infant and other young donors for PDEK but noted that many surgeons are still hesitant to use such tissue for DMEK because peeling of the DM is technically challenging and risks tissue loss. Moreover, young DMEK tissue is thinner and makes tighter scrolls making opening in the anterior chamber very challenging. Their young-donor series showed significant scar resolution and improved vision, with no detachments observed [12].

Creating a reliable type 1 BB is crucial for consistent PDEK grafts; however, bubble behaviour can differ based on the technique and tissue processing. Many authors have introduced new tools or refined techniques to achieve the type 1 BB. In 2016, Dua et al. developed the PDEK clamp, a surgical tool with a spring handle, rings, and a locking screw to seal peripheral fenestrations, preventing air escape and promoting a type 1 BB [7]. Pereira et al. also described a technique to avoid air access to the plane in front of the DM by circumferentially scoring the DM along the stromal edge, creating an escape route for air and ensuring a 100% formation of type 1 BB [26]. To prevent type 2 BB risk, Saint-Jean et al. in 2019 introduced the Soper technique, involving 360° scoring of the DM and endothelium near the Schwalbe line and incremental air injection for an escape route [16]. In 2022, Agarwal et al. used i-OCT to guide donor preparation, assessing needle depth and bevel orientation before air injection [32]. Recently, Kalinnikov et al. proposed a system with a dedicated base, ring fixator, and spring-loaded syringe [6]. In 2019, Tsatsos et al. presented evidence from eye-bank studies indicating that the choice of injectate influences the bubble plane: in a sample of 40 corneas, pneumatic dissection with air resulted in type 1 BB in approximately 92%, whereas switching to fluid reduced type 1 occurrences to approximately 69% and produced all mixed (type 3) bubbles. Consequently, they recommend initially using air and reserving fluid as an alternative, acknowledging that this shift in injectate alters the outcomes towards types 2 and 3 [15]. Besides correct type 1 BB formation, a key challenge in PDEK remains postoperative graft detachment, which continues to be a significant cause of secondary interventions after PDEK. To avoid this issue, Jacob et al. introduced new stabilisation methods utilising the host’s DM and wound scaffolding. They secured the graft by partially overlapping it with the edge of the host DM or the inner lip of the corneal incision. By placing the donor tissue between layers of the host, these techniques offered extra stability and enhanced graft attachment [29]. The safety of PDEK graft preparation has been investigated in Ex vivo studies, particularly in relation to ECL. Altaan et al. conducted a comparison of pneumodissection techniques for PDEK and DMEK grafts, reporting a significantly lower mean ECL with PDEK (5.36 ± 3.8%) compared to DMEK (12.44 ± 8.11%). They discovered that incorporating the PDL/DL improves mechanical stability and safeguards the endothelium during graft preparation [14]. In a subsequent study, Bedard and Hou examined eye bank data and demonstrated that the ECL in PDEK frequently exhibits a reticular pattern localised to the region of bubble formation, with elevated peak inflation pressures correlated with an increased risk of ECL ≥ 25%. They found that as few as 36.6% of grafts could be prepared successfully with minimal ECL or perforation, and emphasised the technical challenge and reproducibility of PDEK preparation [27].

## Advantages of PDEK: clinical and surgical perspectives

Despite being a relatively recent addition to EK, many studies have highlighted the benefits of PDEK in patients undergoing EK. Nowadays, PDEK is a valuable addition to EK techniques, with several advantages over DSAEK and DMEK.

Most of the benefits are associated with the anatomy of the PDL/DL, graft preparation and handling, and its adaptability to different surgical approaches [37].

Due to its anatomy, PDEK maintains all the benefits of the thinnest Descemet stripping endothelial keratoplasty (DSEK) and addresses some of the challenges associated with DMEK, particularly the learning curve. In addition, regarding graft preparation, while Ultra-Thin (UT)-DSEK requires a complex and costly microkeratome to perform a “double-pass” cut for creating the thin corneal lenticule, PDEK tissue can be obtained with minimal tools, enabling broader clinical use and global application [7, 38].

Various surgical PDEK techniques have been proposed, all of which depend on the premise that the PDL/DL splints DM and that the scroll formed is looser than when only DM is involved. As a result, it unrolls effortlessly, as shown by Dua et al., who found that the low scrolling tendency demonstrated by PDL and the high tendency demonstrated by DM suggest that the DM drives most of the scrolling observed in PDEK tissue. Therefore, the addition of PDL/DL provides greater thickness and mechanical stability to the graft compared with the delicate DM alone in DMEK. As a result, PDEK grafts are less prone to tearing, scrolling, or folding irreversibly, which reduces intraoperative graft loss. Therefore, due to its natural tendency to unfold less tightly, the donor lenticule can be easily unfolded in the AC with minimal manipulation [9]. Additionally, the presence of PDL/DL allows for different donor preparation techniques such as air-pump-assisted separation, i-OCT guidance, or PDEK clamps, which have been shown to achieve consistent Type 1 bubble formation and reproducible graft quality [13, 17, 19]. A key feature of PDEK is that it expands the donor pool by including young donors, who are often avoided for DMEK due to challenges in detaching the DM from the residual cornea and the tendency for the tissue to scroll during harvesting tightly. Consequently, many surgeons avoid using young donor tissues to prevent wastage, especially in regions with limited donor availability.

Furthermore, PDEK has demonstrated its versatility in challenging clinical cases [22, 30].

Successful results have been documented in conditions such as PBK, failed PK cases, and even among patients with concomitant endothelial dysfunction and inadequate posterior capsule support. Several surgeons have combined PDEK with various surgeries, including phacoemulsification, SFT pupilloplasty, and scleral fixation IOL, with satisfactory outcomes. Indeed, the reduced surgical complexities, improved ease of tissue handling, and decreased rates of rebubbling associated with PDEK may favour its selection in challenging cases involving anatomically or functionally unicameral eyes, such as those with aphakia, prior trabeculectomy, vitrectomy, or large iris defects, as well as in eyes presenting with extensive peripheral anterior synechiae, scleral-fixated or anterior chamber intraocular lenses, or poor visibility of the anterior segment due to thicker corneas [5, 21, 25, 33, 34].

## Future directions

Although PDEK demonstrates promising anatomical and functional results, its widespread adoption is still limited. Additional time is needed to enable wider dissemination, including gathering long-term data and creating thorough training programs. Looking ahead, long-term, multicentre, prospective studies with extended follow-up will be essential to verify the enduring success of PDEK. As surgical training programs begin to incorporate the new instruments outlined in this review and as standard algorithms develop, PDEK may progress from a niche option to a more widely adopted technique within the spectrum of EK. Since 2013, the PDL/DL discovery has revolutionised the field of lamellar corneal transplantation, and PDEK has now become an integral part of this ongoing transformation [4]. Since its inception, several surgical options have been explored, yielding successful and promising outcomes, even in complex surgeries that require multiple surgical interventions. The significance of PDL/DL anatomy in the history of PDEK is unequivocal; however, the role of young endothelial cells in stromal clearing and regeneration remains to be fully explored. The remarkable clearance of grossly opaque stroma with the use of young donors, as is possible with PDEK, and as has been demonstrated, will gain more attention and analysis. The role of elastin content and distribution in the two PDEK tissues, PDL and DM, needs to be elucidated, as this can impact DMEK, understanding of the mechanism of attachment/apposition of DM to the PDL, and the pathogenesis of some posterior corneal diseases.
